# Inhibition of JAK2/STAT3/SOCS3 signaling attenuates atherosclerosis in rabbit

**DOI:** 10.1186/s12872-020-01391-7

**Published:** 2020-03-13

**Authors:** Xilan Yang, Jian Jia, Zhen Yu, Zheng Duanmu, Huiwei He, Sen Chen, Chen Qu

**Affiliations:** 1 The Second Affiliated Hospital of Nanjing Medical University, Nanjing, 210011 China; 2 The First Affiliated Hospital of Nanjing Medical University, Nanjing, 210029 China; 3 Beijing Information Science & Technology University, Beijing, 100192 China

**Keywords:** JAK2/STAT3/SOCS3 signaling pathway, Atherosclerosis, Ruxolitinib

## Abstract

**Background:**

Previous studies have indicated that the JAK/STAT signaling pathway is involved in modulating arterial adventitia inflammation response. In this study, we designed experiments to further investigate the effect of JAK2/STAT3/SOCS3 signaling in rabbit atherosclerosis process.

**Methods:**

Atherosclerosis was induced in the abdominal arteries of rabbits by balloon injury of the aorta supplemented by the atherogenic diet. Simultaneously, in the process of atherosclerosis, animals underwent either ruxolitinib treatment or not for 12 weeks. At the end of the experimental period, all rabbits were sacrificed. The plaque areas in abdominal artery, the lipid burden of plaque and the calcium burden of plaque were detected by H&E staining, Oil Red O staining and Alizarin Red staining, respectively. In addition, rabbit plasma lipids and inflammatory cytokines were measured by biochemical test kits or ELISA kits. Finally, the expression and phosphorylation levels of JAK2/STAT3/SOCS3 pathway-related proteins were detected by RT-qPCR, western blot and immunohistochemistry assays.

**Results:**

H&E staining and CT scan analysis showed that rabbit atherosclerosis model was constructed successfully. Ruxolitinib, an inhibitor of the Janus kinase 2 (JAK2), substantially reduced the area of atherosclerotic plaques in rabbits treated with high fat diet and balloon injury of the aorta. Moreover, ruxolitinib significantly decreased IL-6, IL-1β, IFN-γ and TNF-α, but increased IL-10 and IL-17 levels in plasma of atherosclerotic rabbits. Additionally, ruxolitinib reduced plasma TC, TG and LDL-C contents and AIP value, while enhanced HDL-C level in atherosclerotic rabbits. Furthermore, we found that JAK2 and STAT3 phosphorylation were up-regulated in rabbits with atherosclerosis when compared with those of the control group, followed by the expression of SOCS3 was also increased due to the activation of JAK2 and STAT3. Interestingly, ruxolitinib could inactivate JAK2 and STAT3 pathway and decrease SOCS3 expression.

**Conclusion:**

Taken together, the inhibition of JAK2/STAT3/SOCS3 signaling pathway may be a novel method for the clinical treatment of artery atherosclerosis.

## Background

Atherosclerosis, a complex cardiovascular disease, has been reported as a chronic inflammatory disease by the increasing studies [1, 2]. At different stages of atherosclerosis, the infiltration of various inflammatory cells, such as T cells, mast cells and macrophages, into the atherosclerotic plaques is one of the main characteristics of atherosclerosis [3]. Subsequently, the migration and proliferation of vascular smooth muscle cells (VSMCs), which attribute to the formation of neointima and atherosclerotic plaques, could be promoted by these infiltrated inflammatory cells in company with the resident vascular wall cells via the secretion of cytokines and growth factors [4–6].

As reported in previous studies, in the process of atherosclerotic lesion development Janus kinase/signal transducer and activator of transcription (JAK/STAT) signaling pathway play a key role [7–9]. Suppressor of Cytokine Signaling 3 (SOCS3) can negatively regulate cytokine signaling by inhibiting JAK/STAT signaling pathway and then exert profound actions in regulating immunity and inflammation [10]. The specific JAK1/2 inhibitor--ruxolitinib is used to treat myelofibrosis and has been approved by FDA [11]. However, whether ruxolitinib plays a key role in atherosclerosis process and JAK2/STAT3/SOCS3 signaling pathway is still not well understood.

In our study, we are committed to explore the underlying role of ruxolitinib on atherosclerosis progression. Interestingly, we found that the area of atherosclerotic plaques was substantially decreased by ruxolitinib in rabbits treated with high fat diet and balloon injury of the aorta. Moreover, ruxolitinib remarkably decreased plasma levels of IL-6, IL-1β, IFN-γ and TNF-α in rabbits with atherosclerosis. Differently, the levels of plasma IL-10 and IL-17 were significantly increased in ruxolitinib-treated atherosclerotic rabbits. Furthermore, we found that ruxolitinib inactivated JAK2 and STAT3 pathway and decreased SOCS3 expression. From those results, we finally concluded that the inhibition of JAK2/STAT3/SOCS3 signaling may attenuate atherosclerosis in rabbits.

## Methods

### Animals

Ten New Zealand male rabbits, weighted 3.4 ± 0.6 kg, were purchased from Qing Long Shan Dong Wu Fan Zhi Chang (Nanjing, China). Rabbits were randomly assigned to three analyzed groups: Control (normal diet, no ruxolitinib, *n* = 3); Model (balloon injury of the aorta and high fat diet, *n* = 3); and ruxolitinib (balloon injury of the aorta and high fat diet with the addition of ruxolitinib, *n* = 4). Aortic atherosclerotic plaques were induced in rabbits by high fat diet and repeated balloon injury of the aorta. Aortic injury was performed from the aortic arch to the iliac bifurcation with a 4-French Fogarty embolectomy catheter (Edwards Lifesciences) introduced through the femoral artery. All procedures were performed under general anesthesia induced by Pentobarbital (30 mg/kg) and euthanized by exsanguination. All experiments were approved by the Second Affiliated Hospital of Nanjing Medical University of Medicine Institute Animal Care and Use Committee.

### CT protocol

A dual-source CT scanner (Somatom Definition; Siemens Medical Solutions, Forchheim, Germany) was used to perform the CT examination as described previously [12]. To ensure the entire thorax and abdomen of animals were covered by the field of view of the larger and smaller tube detector arrays, all rabbits were centrally placed in the scanner with the field of view of the second tube detector array was 260 mm. For the initial unenhanced chest CT scanning, the testing parameters were set as Zhang et al. reported [12]. Hereinto, the weighted CT dose index (CTDIw) was 4–5 mGy, which was calculated according to the equation as follows: CTDIw = CTDIvol × P, where CTDIvol was volume CT dose index and P was pitch. After the injection of iopromide (300 mg/mL) with the injection dose of 2 mL/kg at a rate of 1.8 mL/sec. Subsequently, a 10-mL saline solution was injected into an internal jugular vein via an 18-gauge catheter prior to contrast-enhanced CT with the dual-energy mode. A bolus-tracking technique was used to trigger the CT scan and the image acquisition of the region of interest placed in the abdominal aorta started 3 s later once the attenuation up to the predefined threshold of 100 HU. In addition, the other CT scanning parameters were listed following: tube voltages: 80 and 140 kVp; tube currents: 183 mA for the larger x-ray tubes and 51 mA for the smaller x-ray tubes; gantry rotation time: 0.33-s; detector collimation: 14 × 1.2-mm, pitch: 0.5; field of view: 260-mm.

### Sample collection

Animals were anesthetized with an overdose of pentobarbital for abdominal aorta obtain. After carefully peeling off the adventitial layer, a small transverse cut was made partially through the iliac artery (about 3 mm past the bifurcation) to split the vessel and the cut proceeds anteriorly until the kidneys were reached. The entire abdominal aorta was then excised and cut into four segments. The aorta samples stored at − 80 °C were used for protein expression studies and frozen section preparation; the aorta samples fixed with 4% formaldehyde were prepared for the routine Hematoxylin-Eosin staining; the aorta samples immediately snap-frozen in liquid nitrogen were adopted for RT-qPCR assay. In addition, at the beginning of the study, the moment of randomization and the end of the experiment, rabbit blood samples were respectively collected through a vein in the ear edge at 24-h post-meal.

### Histology and lesion analysis

For histology, abdominal aorta samples were fixed with 4% formaldehyde and embedded in paraffin. Then, the paraffin-embedded tissues were cut into 4 μm slices, and the sections were stained using Hematoxylin and Eosin Staining Kit (Beyotime Institute of Biotechnology, Shanghai, China). Finally, all sections were scanned at an absolute magnification of 100× under a light microscope (Olympus, BX51 microscope, Tokyo, Japan). All changes in blood vessel layers were observed and photographed. The average aortic plaque area derived from the H&E stained histology were measured by image-pro plus (IPP) software (Media Cybernetics, Inc.). In addition, Oil Red O staining was performed for quantification of lipid burden in plaques. Briefly, the aorta samples stored at − 80 °C were processed into serial frozen sections and stained by 0.5% Oil Red O (Wako, Osaka, Japan). Positive areas of Oil Red O staining in the lesions were determined using WinROOF ver.6 (Mitani Corporation, Tokyo, Japan) as described previously [13]. Further, Alizarin Red S staining was used to detect calcium deposition. The frozen sections were fixed in 95% ice-cold ethanol and then stained by 2% Alizarin Red S (pH 4.2, Sigma) at room temperature for 15 min. The emergence of mineralized nodules was considered as positive.

### Measurement of plasma IL-6, IL-10, IL-17, IL-1β, IFN-γ and tumor necrosis factor (TNF)-α

The collected blood samples were centrifugated at 2000×g for 15 min; then plasma was separated and stored at − 80 °C until use. The contents of plasma IL-6, IL-10, IL-17, IL-1β, IFN-γ and TNF-α were all measured with enzyme-linked immunosorbent assay kits (MILENIA BIOTEC, Gmbh, Germany) according to the manufacturer’s protocol.

### Measurement of plasma TC, TG, LDL-C and HDL-C

At the end of the experiment, blood was collected from the ear-vein of a rabbit with heparin anticoagulation. The plasma was separated by frozen centrifugation for 10 min at 6000×g. The contents of total cholesterol (TC), triglyceride (TG), low-density lipoprotein cholesterol (LDL-C) and high-density lipoprotein cholesterol (HDL-C) were measured using biochemical test kits (Nanjing Jiancheng Bioengineering Institute, Nanjing China) with the semi auto analyzer by colorimetric method.

### Atherogenic index plasma (AIP)

AIP, which is the logarithmic transformation of the plasma triglyceride (TG) level to the high-density lipoprotein level (HDL) ratio, is the measurement of the atherosclerotic lesion extent based on plasma lipids and can act as a better marker than the TC/HDL ratio for the detection of subclinical atherosclerosis in AS patients [14, 15].

### Immunohistochemistry assay

The paraffin-embedded tissues were cut into 4 μm slices. Then, the sections were deparaffinated, hydrated and incubated with the primary antibodies at 4 °C overnight. After washing in TBST (Tris-HCl buffer + 0.1% Tween 20), the sections were continually incubated with a secondary antibody labeled with HRP. For immunohistochemistry studies, the stain was visualized by 3, 3-diaminobenzidine (DAB) and Harris hematoxylin and analyzed under an Olympus microscope.

### Western blot

The aorta samples stored at − 80 °C were homogenized in the RIPA lysis buffer, and protein concentration was quantified by BCA Protein Assay Kit (Beyotime Institute of Biotechnology, Shanghai, China). The protein samples were then separated by SDS-PAGE and transferred onto PVDF membrane. The membranes were blocked with 5% BSA (5% w/v in PBS + 0.1% Tween 20) and incubated with primary antibodies at room temperature. Antibodies against p-JAK2, JAK2, p-STAT3, STAT3, SOCS3, and β-actin were used according to the manufacturer’s instructions, and were purchased from Santa Cruz Biotechnology (Santa Cruz, CA). The membranes were washed in PBS containing 0.03% Tween 20 and further incubated with a peroxidase-conjugated secondary antibody for 1 h. After washing, the membranes were detected by SuperSignal West Pico Chemiluminescent Substrate kit (Pierce, Rockford, USA) according to the manufacturer’s instructions.

### Real-time PCR

Frozen aorta samples were ground in liquid nitrogen for total RNA extraction using Trizol reagents. Then, cDNA samples were synthesized by the HiFiScript cDNA Synthesis Kit (Cwbio, China). After identifying purity and integrity, the cDNA samples were quantified and served as the template to measure the expression of SOCS3 mRNA using Greenstar qPCR Master Mix (Bioneer, South Korea). Primer sequences used for quantitative RT-PCR were as following: β-actin 5′-CAT CAC CAT TGG CAA TGA GC-3′ and 5′-TCG TCA TAC TCC TGC TTG C-3′; SOCS3 5′-AGT TCC TGG ACC AGT ACG A-3′ and 5′-TTC CTC CAC ACT GGA TTC TTG-3′. The 2^-ΔΔCt^ method was used for calculation of SOCS3 mRNA with β-actin gene as a reference.

### Statistical analysis

Data are presented as mean ± SD of three independent experiments. Differences between groups were analyzed by GraphPad Prism 5 software (GraphPad Software, CA, USA) with Student’s t-test. Differences were considered statistically significant at *P < 0.05*.

## Results

### Establishment of rabbit atherosclerosis model

**Fig. 1:**
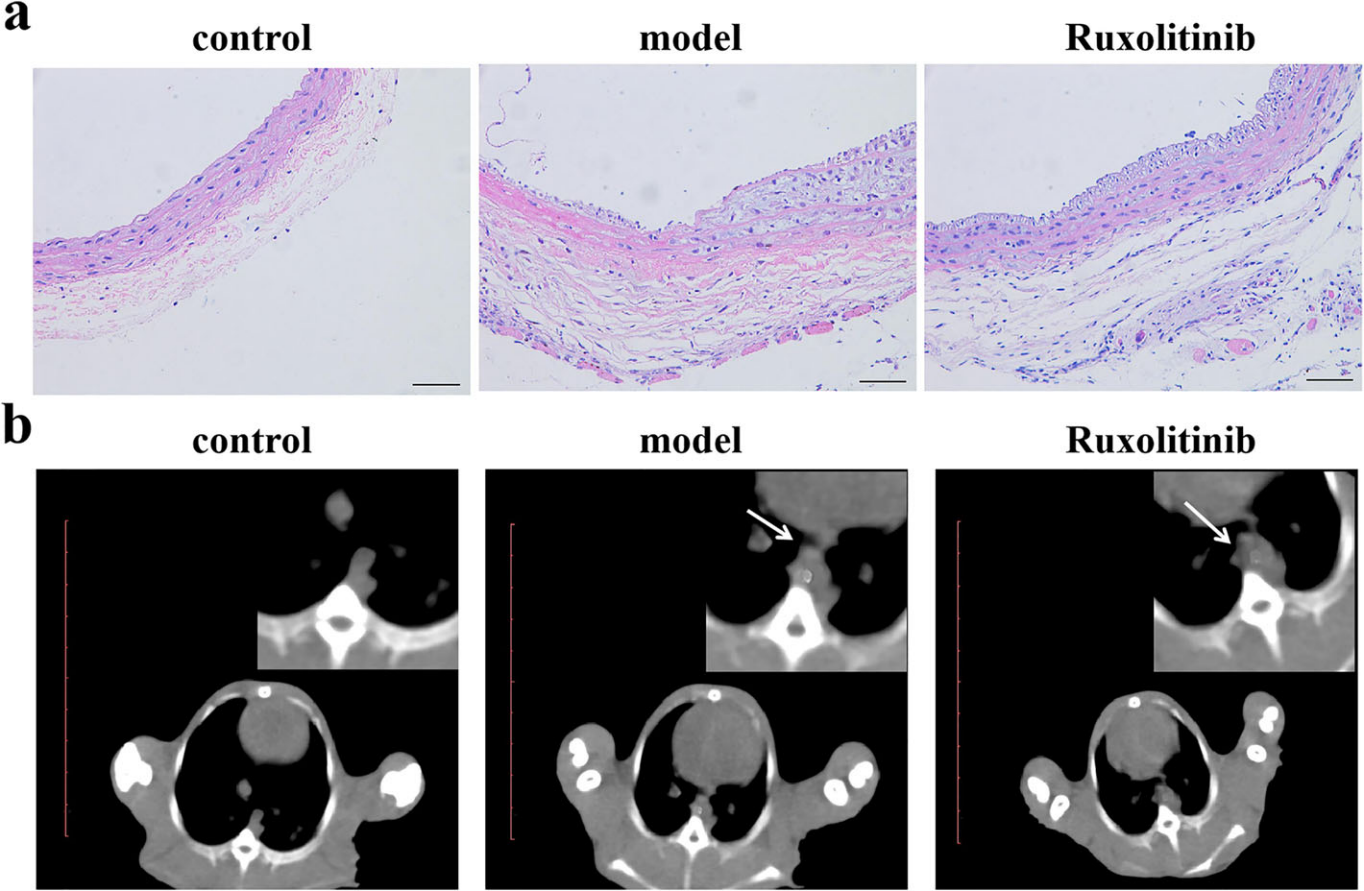
Ruxolitinib alleviated atherosclerosis progress in rabbits. (**a**) Representative images of H&E staining of abdominal aorta sections (100×). (**b**) Representative images of CT scan images of abdominal aorta sections. The upper right corner: magnified CT images which point out the areas of lesions (marked by the white arrow)**.** Scale bar, 1 cm

**Fig. 2:**
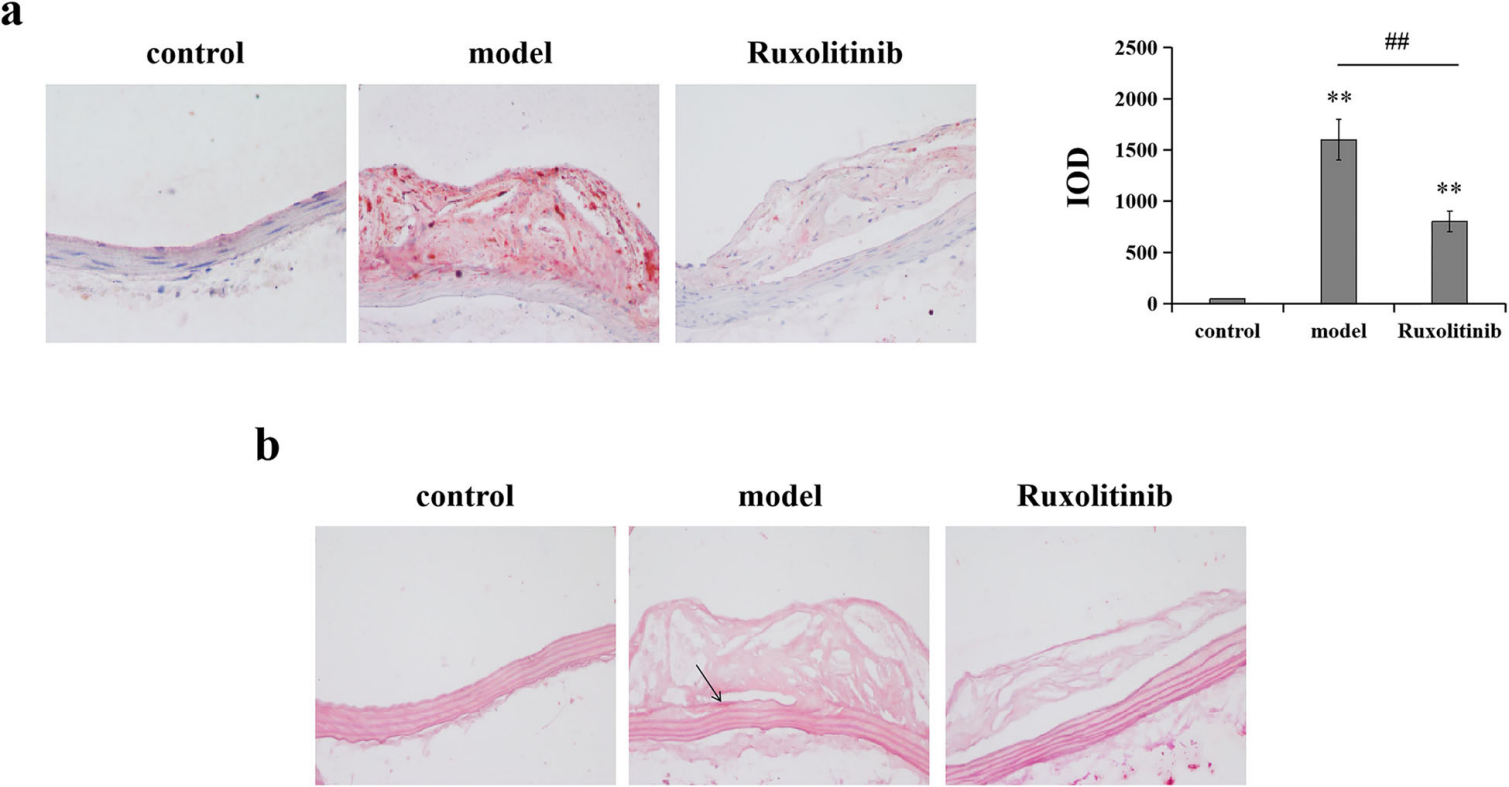
Ruxolitinib decreased the lipid burden and calcium burden in plaques of atherosclerotic rabbits. **(a)** Oil Red O staining for the deposition of lipid droplets. Dark-staining red drops: positive areas of Oil Red O staining; quantification determined by WinROOF ver.6 (right). ^**^P < 0.01 versus control group; ^##^*P* < 0.01 versus model group. Data were analyzed by using Student’s t-test and expressed as mean ± SD (*n* = 3). (**b**) Alizarin Red S staining for calcium burden in plaques. The emergence of mineralized nodules (Dark-staining red areas, marked by the black arrow) was considered as positive

To verify whether the rabbit atherosclerosis model was constructed successfully, rabbit atherosclerotic plaques were detected by H&E staining, CT scan analysis, Oil Red O and Alizarin Red S staining. In this study, representative images of H&E staining showed that the area of atherosclerotic plaques was obviously increased in rabbits with high fat diet and aortic intimal injury when compared with the control group (Fig. 1a). CT scan analysis also displayed obvious atherosclerotic imaging area (Fig. 1b, marked by the white arrow). In addition, from the results from Oil Red O and Alizarin Red S staining (Fig. 2), we also found that rabbits underwent high fat diet and aortic intimal injury exhibited obvious lipid burden and modest calcium burden (red staining area, marked by black arrow) in atherosclerotic plaques, which indicated that the rabbit atherosclerosis model was constructed successfully.

### Ruxolitinib alleviated atherosclerosis progress in rabbits

It has reported that the JAK/STAT signaling pathway is important in atherosclerotic lesion development [7, 9, 16], so we next explored the function of ruxolitinib, an inhibitor of JAK2, in atherosclerosis progress of rabbits. In this study, we found that the area of atherosclerotic plaques was obviously decreased after feeding with ruxolitinib in rabbits with high fat diet when compared with that of the model group. Similarly, the aortic intimal injury was also alleviated by ruxolitinib as the results from H&E staining and CT scan analysis (Fig. 1a and b). In addition, no mineralized nodules (dark-staining red area) were observed in the abdominal aorta from ruxolitinib-fed atherosclerotic rabbits, suggesting that ruxolitinib may act as an inhibitor in the rabbit atherosclerosis progress.

### Ruxolitinib reduced plasma lipids levels and plaques lipid burden in atherosclerotic rabbits

**Fig. 3:**
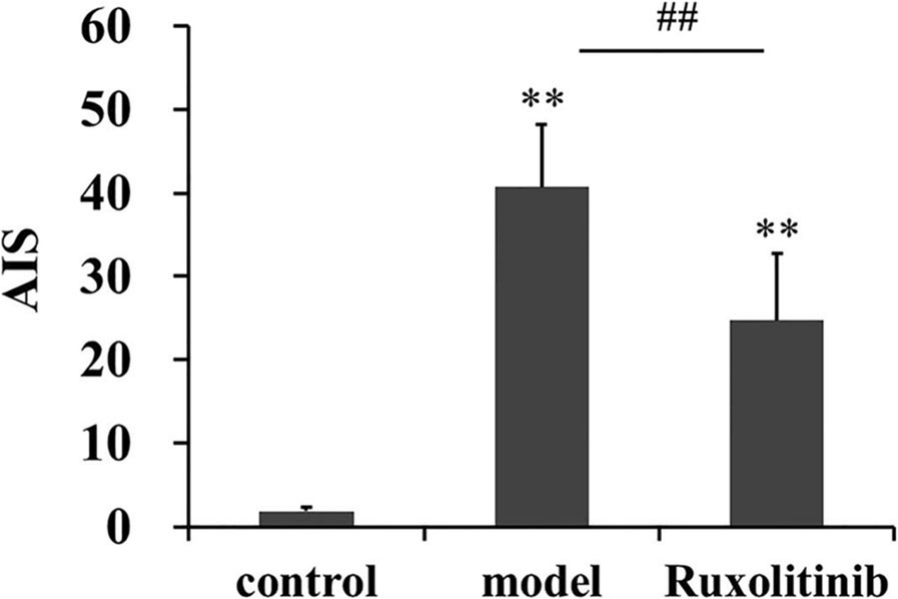
Ruxolitinib reduced AIP value in atherosclerotic rabbits. AIP was the measurement of the atherosclerotic lesion extent based on plasma lipids. ^**^P < 0.01 versus control group; ^##^*P* < 0.01 versus model group. Data were analyzed by using Student’s t-test and expressed as mean ± SD (*n* = 3)

It is generally known that the lipid levels in the blood are an important index to assess atherosclerosis progress [17]. As expected, the levels of plasma TG, TC and LDL-C were significantly increased, while HDL-C level was decreased after 12 weeks of high fat diet feeding. Interestingly, ruxolitinib could regulate the abnormal lipid levels, reflected as the significantly decreased TG, TC and LDL-C contents but increased HDL-C content when compared with those of normal control group (Table. 1, *p* < 0.05). Additionally, obviously enhanced AIP in model group could also be reduced by ruxolitinib (*P* < 0.05, Fig. 3). Similarly, the results from Oil Red O staining also showed that some lipid droplets could be observed in plaques in atherosclerotic rabbits, however ruxolitinib could reduce the lipid burden in plaques to some degree (^##^*p* < 0.05), indicating that ruxolitinib may reduce plasma lipids levels and plaques lipid burden in the process of atherosclerosis.

**Table 1:** Ruxolitinib regulated TC, TG LDL-C and HDL-C levels in rabbit plasmas (mean ± SD, *n* = 3 or 4)

Groups	TC (mmol/L)	TG (mmol/L)	LDL-C (mmol/L)	HDL-C (mmol/L)
Control	2.68 ± 0.41	0.83 ± 0.34	0.23 ± 0.12	1.48 ± 0.30
Model	25.24 ± 2.22^**^	2.44 ± 1.11^**^	1.82 ± 0.35^**^	0.64 ± 0.13^**^
Ruxolitinib	21.15 ± 2.56^**#^	1.64 ± 0.75^*#^	1.02 ± 0.29^**##^	0.91 ± 0.23^*#^

### Ruxolitinib decreased plasma IL-6, IL-1β, IFN-γ and TNF-α levels while increased IL-17 and IL-10 levels

**Fig. 4:**
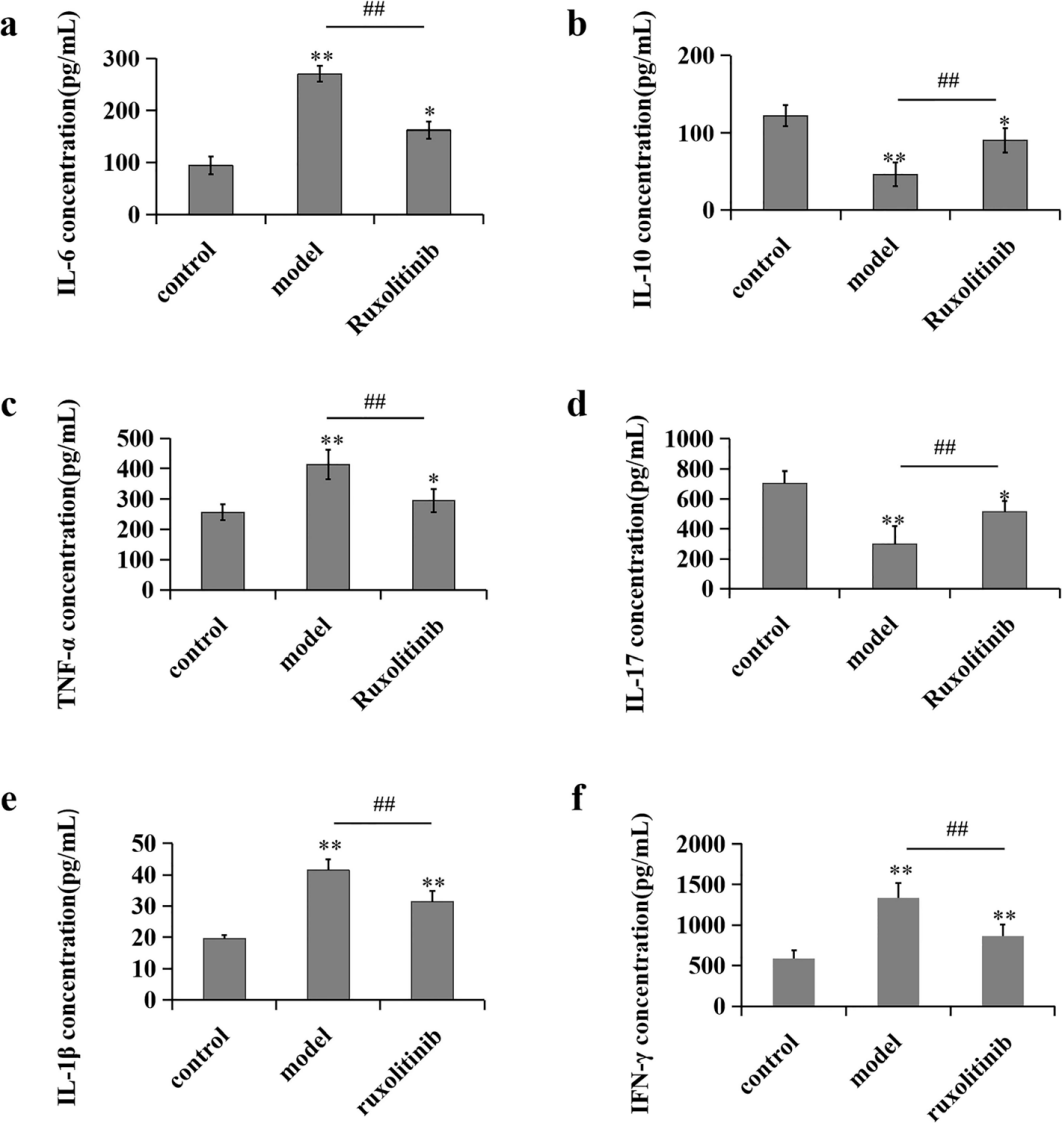
Administration of ruxolitinib decreased the contents of plasma IL-6, TNF-a, IL-1β, IFN-γ, IL-10 and IL-17 in rabbits with atherosclerosis. **(a~f)** The levels of plasma IL-6, IL-1β, IFN-γ, TNF-a, IL-10 and IL-17 in rabbits were detected by enzyme-linked immunosorbent assay kits. ^**^P < 0.01 versus control group; ^##^*P* < 0.01 versus model group. Data were analyzed by using Student’s t-test and expressed as mean ± SD (*n* = 3)

IL-6, a crucial inflammatory stimulus, can induce JAK activation and STAT3 phosphorylation through forming a complex with IL-6 receptor α and gp130. Importantly, TNF-α and IL-1β can promote IL-6 secretion through NF-κB activation [18]. Therefore, in this study, we also detected the contents of plasma IL-6, IL-10, IL-17, IL-1β, IFN-γ and TNF-α by ELISA assay. The results showed that rabbits with atherosclerosis presented higher IL-6, IL-1β, IFN-γ and TNF-α levels than those in the control group; however, ruxolitinib substantially decreased IL-6, IL-1β, IFN-γ and TNF-α contents in rabbits with atherosclerosis. On the contrary, the levels of IL-10 and IL-17 were significantly decreased after balloon injury of the aorta and high fat diet, however ruxolitinib could reverse this decrease (Fig. 4). Data above demonstrated that the JAK inhibitor, ruxolitinib, may mitigate the inflammatory response during atherosclerosis.

### Ruxolitinib alleviated atherosclerosis through the inhibition of JAK2/STAT3/SOCS3 signaling

**Fig. 5:**
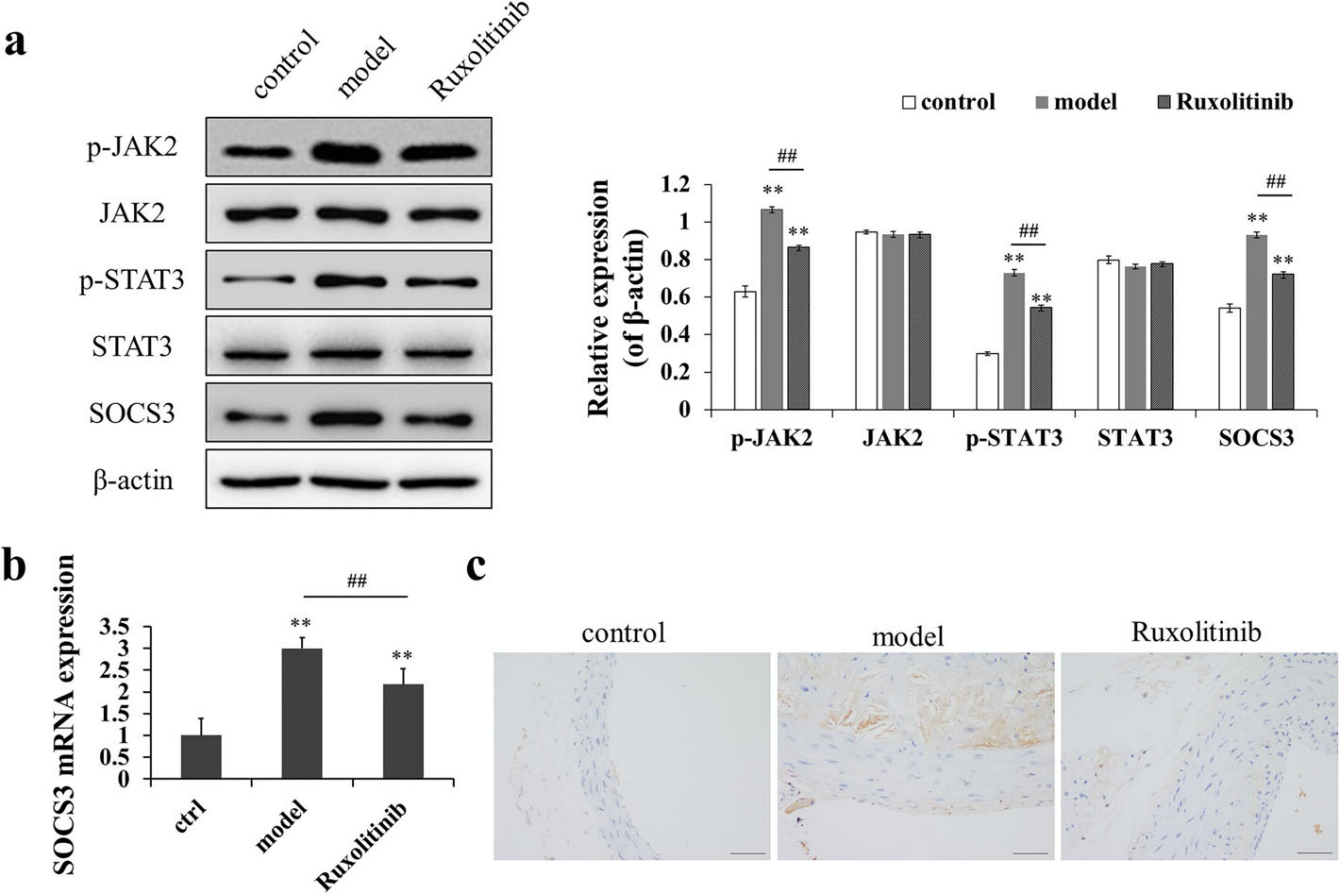
Administration of ruxolitinib inactivated JAK2/STAT3/SOCS3 pathway in rabbit aorta tissues. (**a**) Protein expression levels of p-JAK2, JAK2, p-STAT3, STAT3 and SOCS3 in rabbit aorta tissues by western blot analysis; quantification (right). (**b**) RT-qPCR for SOCS3 expression at mRNA level in rabbit aorta tissues. (**c**) Immunohistochemical staining of p-SOCS3 in rabbit aorta tissues (Scale bar = 100 μm); ^**^P < 0.01 versus control group; ^##^*P* < 0.01 versus model group. Data were analyzed by using Student’s t-test and expressed as mean ± SD (*n* = 3)

To further explore whether ruxolitinib alleviated atherosclerosis through modulating JAK2/STAT3/SOCS3 pathway, we detected the expression of SOCS3 and the phosphorylation of JAK2 and STAT3, and found that the phosphorylation levels of JAK2 and STAT3 were significantly up-regulated in rabbits with atherosclerosis (a two-fold change, *p* < 0.05). Moreover, the expression level of SOCS3 mRNA was also increased due to the activation of JAK2/STAT3 pathway; however, ruxolitinib inactivated JAK2 and STAT3 phosphorylation and decreased SOCS3 expression (Fig. 5a). Additionally, RT-PCR results also demonstrated that the expression of SOCS3 mRNA was significantly down-regulated by ruxolitinib (Fig. 5b). Further, immunohistochemistry assay displayed that there was a high expression level of p-SOCS3 in the atheromatous plaque, reflected as the strong brown-yellow stained areas. Instead, after treatment with ruxolitinib, a significant decrease in the expression of p-SOCS3 was achieved in atheromatous plaque (Fig. 5c). Taken together, our findings suggest that the inhibition of JAK2/STAT3/SOCS3 signaling may attenuate atherosclerosis in rabbits.

## Discussion

Atherosclerosis, one of the leading causes of cardiovascular morbidity and mortality, is a chronic inflammatory vascular disease [19]. More and more evidences have indicated that not only the formation and progression but also the rupture of atherosclerotic plaques could be drove by inflammation [20]. Thus, it will provide novel treatment strategies against atherosclerosis by focusing on the potential targets which play key roles in the inflammatory process in atherosclerosis. The activation of JAK2/STAT3 pathway has been observed in atherosclerotic lesions and the activated JAK2/STAT3 signaling has been proved to play important roles in regulating the cellular activation, proliferation and differentiation of VSMCs and other vascular cells in the occurrence and development of atherosclerosis [21–23]. Our results indicated that high-fat diet and balloon injury of the aorta could cause the occurrence of atherosclerosis and the atherosclerotic rabbit exhibited elevated JAK2 and STAT3 phosphorylation levels, suggesting that the JAK/STAT3 signaling pathway is activated during atherosclerosis.

Hyperlipidemia, one of the most important risk factors account for the further development of atherosclerosis, can activate the JAK/STAT signaling of vascular endothelial cells through phosphorylation of JAK2 and subsequently STAT3, leading to the deterioration of atherosclerosis [24]. AIP acts as a critical mark of atherosclerosis because the deposition of foam cells or plaque or fatty in filtration or lipids in organs could be indicated by it [25]. In this study, a 12-week high-fat diet feeding accompanied by balloon injury of the aorta not only resulted in the increase in TG, TC and LDL-C contents and AIP value, but also led to the obvious lipid burden in atherosclerotic plaques when compared with the normal control rabbit. As expected, ruxolitinib feeding could significantly reduce plasma TG, TC, LDL-C contents and AIP value, but enhanced HDL-C level, suggesting that the abnormal blood lipids could be improved better in the ruxolitinib treatment group. Lipid metabolism in adipocyte can be mediated by the modulation of the JAK/STAT pathway, as the activated STATs can regulate lipid metabolism directly by influencing the expression of enzymes like AOX and FAS and/or regulating inflammation and anti-inflammation response [26], while SOCS3 inhibits the functioning of leptin and downstream steps in insulin signaling after being expressed by terminal transcription factors STAT3 [27]. Here, ruxolitinib, an inhibitor of JAK1/2, could also inhibit the downstream targets (STAT3 and/or SOCS3) of JAK2, reflected as the down-regulated expression of p-STAT3 and SOCS3. That is, ruxolitinib may influence plasma lipid levels in atherosclerotic rabbit by modulating the JAK/STAT pathway and the expression of SOCS3. However, the molecular mechanisms underlying ruxolitinib-mediated lipid metabolism still need further study.

Pro-inflammatory cytokines play a key pathogenic role in atherosclerosis, which are induced by hyperlipidemia and JAK/STAT pathway activation. IL-6, which is highly expressed in atherosclerotic aortas, can directly activate gp130/JAK/STAT3 signaling to exacerbate atherosclerosis [28, 29]. Meanwhile, growing evidences have confirmed that, some inflammatory cytokines, such as IL-10, IL-1β, IFN-γ and TNF-α, may be responsible for the pathogenesis of atherosclerosis because these cytokines can result in the adhesion of leukocytes to vascular endothelium and foam cell formation [30, 31]. Similarly, the contents of plasma IL-6 and TNF-α were significantly increased in atherosclerotic rabbits in our study. Interestingly, we found that ruxolitinib, a specific JAK1/2 inhibitor which used for myelofibrosis treatment, could substantially reduce plasma IL-6, IL-1β, IFN-γ and TNF-α contents in atherosclerotic rabbits. Notably, circulating levels of IL-10 and IL-17 in atherosclerotic rabbits were significantly decreased. It has reported that enhanced IL-17 production associated with increased IL-10 (regulatory Th17) and reduced IFN-γ will most probably limit lesion development and promote plaque stability [32]. In our study, ruxolitinib could delay the decrease of circulating IL-10 and IL-17 and reduce IFN-γ production. Data above suggest that inflammatory processes play important roles in atherosclerosis, and ruxolitinib could alleviate atherosclerosis through regulating the secretion of inflammatory cytokines.

It is well known that proinflammatory cytokines can activate SOCS-3 and JAK/STAT pathway is also regulated by the SOCS proteins. SOCS3, whose expression level increased continuously along with the elongation of feeding in ApoE^−/−^ mouse aortas, is closely correlated with atherosclerosis progression [33] because it plays a regulatory role in the expression and activation of IL-6, TNF-α, as well as other inflammatory cytokines. That is, SOCS3, as a classic negative regulator of JAK2/STAT3, was up-regulated with the progression of inflammation aggravated by hyperlipidemia [34]. Here, a 12-week high-fat diet feeding in rabbit led to an obvious increase of plasma TG, TC and LDL-C, followed by the up-regulated expression and phosphorylation of SOCS3 in atherosclerotic abdominal aorta. Current therapeutic small-molecule JAK/STAT3 inhibitors, such as tasocitinib, can effectively suppress SOCS3 activity [35]. Similarly, the specific JAK1/2 inhibitor-ruxolitinib could also inhibit the expression and phosphorylation of SOCS3 in atherosclerotic rabbit. Additionally, some researches have demonstrated that IL-17 production was enhanced after SOCS3 deletion in T cells [36, 37]. Consistent with above, ruxolitinib inhibited the expression of SOCS3 accompanied by the increase in IL-17 production.

Taking into account our results, we hypothesized that the high level of plasma lipids, increased the secretion of IL-6, IFN-γ and TNF-α, subsequently activating JAK2/STAT3 to induce and aggravate atherosclerosis. Ruxolitinib could down-regulated the expression of p-JAK2, p-STAT3 and STAT3 to block JAK2/STAT3 pathway activation, subsequently prevented the upregulation of most cytokines, especially IL-6, IFN-γ and TNF-α, which are important proinflammatory factors, and finally reduced the formation of atheromatous plaque and lipid burden in plaques. It suggests that ruxolitinib may attenuate atherosclerosis by inhibiting JAK2/STAT3/SOCS3 signaling pathway, while the detailed underlying mechanisms still need further studies.

## Conclusion

In the present work, we found that the JAK2 inhibitor ruxolitinib may reduce the formation of aortic atherosclerotic plaques and decrease the contents of plasma TG, TC and LDL, while enhance HDL-C level in rabbit with atherosclerosis; meanwhile, ruxolitinib may also regulate the secretion of inflammatory components, such as IL-6, IL-10, IL-17, IL-1β, IFN-γ and TNF-α through the inactivation of JAK2/STAT3 pathway and the downregulation of SOCS3 expression. Considering the important role of JAK2/STAT3/SOCS3 signaling pathway played in the inflammatory process in atherosclerosis, the potentially therapeutic strategy may be expected for the treatment of patients with atherosclerosis.

## Data Availability

All data generated or analysed during this study are included in this published article.

## Abbreviations

AIP: Atherogenic index plasma
CTDIw: Weighted CT dose index
DAB: 3, 3-diaminobenzidine
HDL-C: High-density lipoprotein cholesterol
JAK: Janus kinase
LDL: Low-density lipoprotein
SOCS3: Suppressor of cytokine signaling 3
STAT: Signal transducer and activator of transcription
TC: Total cholesterol
TG: Triglyceride
Th17: T helper 17
TNF: Tumor necrosis factor
